# Impacts of Temperature on the Growth, Feed Utilization, Stress, and Hemato-Immune Responses of Cherry Salmon (*Oncorhynchus masou*)

**DOI:** 10.3390/ani13243870

**Published:** 2023-12-15

**Authors:** Jang-Won Lee, Balamuralikrishnan Balasubramanian

**Affiliations:** 1 Department of Integrative Biological Sciences and Industry, College of Life Science, Sejong University, Seoul 05006, Republic of Korea; 2 Department of Food Science and Biotechnology, College of Life Science, Sejong University, Seoul 05006, Republic of Korea; bala.m.k@sejong.ac.kr

**Keywords:** *Oncorhynchus masou*, temperature, growth, feed efficiency, stress, health

## Abstract

**Simple Summary:**

Cherry salmon (*Oncorhynchus masou*) is a commercially important species found in the Northwest Pacific Ocean. Although it is commercially farmed within its range, limited data exist regarding the precise temperature ranges conducive to growth, efficient feed utilization, and overall health in a controlled environment. This study aimed to characterize the temperatures commonly experienced by the species in land-based aquaculture farms, seeking to identify those that most effectively promote growth, feed utilization, and health. Additionally, it sought to investigate the repercussions of less conducive temperatures on stress levels, osmoregulatory capabilities, and immune responses. We found that the water temperatures, within the temperatures tested, most conducive to growth, feed efficiency, and health were 10 °C and 14 °C, while the least favorable temperature was identified at 22 °C. Furthermore, a temperature of 22 °C resulted in delayed feeding response, reduced appetite, compromised health status, impaired osmoregulation ability, and disrupted immune response. Thus, rearing temperatures should be maintained below 22 °C in commercial farms to avoid deterioration in health and well-being, as well as decreases in growth and feed efficiency, of this fish.

**Abstract:**

Cherry salmon (*Oncorhynchus masou*) hold commercial value in aquaculture, and there is a need for controlled laboratory studies to isolate the specific effects of temperature on their growth, feeding, and well-being. We examined the effects of different temperatures (10 °C, 14 °C, 18 °C, and 22 °C) on juvenile cherry salmon (average mass 29.1 g) in triplicate tanks per treatment over eight weeks. The key parameters assessed included growth rate, feed efficiency, stress response, and hemato-immune responses. Our objectives were to determine the most and less favorable temperatures among the four designated temperatures and to assess the adverse effects associated with these less favorable temperatures. The results showed that body weight, growth rates, feed intake, and feed efficiency were significantly higher at 10 °C and 14 °C compared to 18 °C and 22 °C. Reduced appetite and feeding response were observed at 22 °C. Red blood cell parameters were significantly lower at 22 °C. At 10 °C, the results showed significantly increased plasma cortisol levels, gill Na^+^/K^+^-ATPase activity, body silvering, and decreased condition factors, suggesting potential smoltification. The potential smoltification decreased with increasing temperatures and disappeared at 22 °C. Furthermore, the plasma lysozyme concentrations significantly increased at 18 °C and 22 °C. In conclusion, our study identifies 10 °C and 14 °C as the temperatures most conducive to growth and feed performance in juvenile cherry salmon under these experimental conditions. However, temperatures of 22 °C or higher should be avoided to prevent compromised feeding, reduced health, disturbed immune responses, impaired growth, and feed performance.

## 1. Introduction

Water temperature is a critical environmental factor that influences every aspect of physiological activities, including stress response, feeding, and growth, in fish [1]. The significance of investigating temperature effects, especially elevated temperature in cold-water species like salmonids, is evident in the wealth of accumulated literature. For instance, Brett et al. [2] found that sockeye salmon (*Oncorhynchus nerka* Walbaum) exhibited optimal growth at 15 °C, with no growth observed around 23 °C, despite surplus ration. Marine and Cech [3] showed that rearing temperatures within the range of 21–24 °C affected the growth rates, smoltification indices, and predation avoidance of juvenile Chinook salmon (*Oncorhynchus tshawytscha*), while 17–20 °C primarily impacted the latter two parameters compared to 13–16 °C. Additionally, Steiner et al. [4] reported that both growth and feed intake (FI) increased with rising temperatures up to 20 °C in Chinook salmon. For Atlantic salmon (*Salmo salar*), Koskela et al. [5] found that the optimal temperatures for feed intake and growth were 17.8 °C and 15.6 °C, respectively, with estimated upper thermal limits for these parameters at 29.0 °C and 24.1 °C. Similarly, Hvas et al. [6] reported that Atlantic salmon reared at 23 °C for 4 weeks experienced poor appetite, significantly lower condition factors, and increased mortality (~20%) compared to those at 18 °C or lower temperatures. While extensive research has explored temperature effects on feeding and growth in anadromous salmonids, including the optimal and upper limits temperatures [2,3,4,5,6,7], the impact of sublethal elevated temperatures on other physiological parameters related to overall health, stress, osmoregulation, and immune response remains to be further investigated [8], particularly in some salmonid species.

Hematological parameters, including hematocrit (Hct), red blood cell (RBC) count, and hemoglobin (Hb) concentration, have been utilized to diagnose the pathophysiological changes of fish in response to environmental changes [8,9]. The RBC parameters can provide an integrated description of the adaptability and/or health of a specific fish species under defined environmental conditions such as temperatures [8]. In some studies, elevated water temperatures have been observed to increase the RBC indices in fish to meet the increased metabolic rate and oxygen demand [1,10,11]. On the other hand, elevated water temperatures have led to a decrease in RBC indices in other studies [12,13,14]. The extent of stress induced by elevated temperatures can lead to either a significant increase or reduction in RBC indices, depending on the magnitude and duration of the temperature changes and the thermal adaptability of the fish [15]. While the analysis of fish RBC indices provides valuable insight into a fish’s adaptability to temperature changes [8,14], it is important to consider additional physiological parameters to gain a more comprehensive understanding of the mechanisms underlying the effects of temperature increases on different fish species.

The capacity of fish to manage environmental changes such as temperature increase could be further elucidated by considering stress physiological parameters, including plasma cortisol and glucose [16,17]. Increases in circulating cortisol levels as a primary stress response are known to be able to mediate secondary and/or tertiary stress responses depending on the extent and duration of stress [17,18,19]. If a certain fish species exhibits an increase in plasma cortisol levels, accompanied by tertiary stress responses such as depressed health status, immune competence, and growth, as a result of chronic exposure to elevated temperatures, it may indicate that the temperature is beyond the species’ physiological optimal range [16,17]. In previous studies, elevated plasma cortisol levels in exposure to acute temperature increases have been reported in various fish, including salmonids [17,20,21]. On the other hand, significant increases in cortisol levels were observed in salmonid fish when chronically exposed to elevated temperatures in some studies [16,22], but not in other studies [19,23,24]. The latter response is possibly related to the negative feedback loop impacting cortisol production, which may be a result of overcoming thermal stress [17]. Thus, plasma cortisol response can provide a picture of the adaptability of a species to a certain temperature increase and help predict secondary responses such as growth rate [16]. However, chronic exposure to elevated temperatures on plasma cortisol levels and the subsequent glucose response is very limited in anadromous salmonids [22].

Cortisol is also well-known as a salt-secreting hormone in euryhaline fish [25,26]. Plasma cortisol is reported to be involved in mediating parr–smolt transformation and hypo-osmoregulation ability in anadromous salmonids [27]. However, the increased temperatures affected plasma cortisol levels and the Na^+^/K^+^-ATPase activity in salmonids [3,28,29]. Measurements of plasma cortisol and gill Na^+^/K^+^-ATPase activity can help in exploring osmoregulation ability at elevated temperatures.

Temperature is known to impact immune function [30,31]. It has been reported that fish generally show high immune competence at physiologically permissive temperatures when compared with that at physiologically non-permissive temperatures [13,32,33,34]. Like most vertebrates, salmonids possess non-specific and specific immune systems, but these systems may differentially function at different rearing temperatures [33]. It could be informative to determine both non-specific and specific immune parameters to explore the relationship between temperature and immune function.

The cherry salmon (also called Masu salmon, *Oncorhynchus masou*, Brevoort, 1856) is exclusively found in the Northwest Pacific Ocean, specifically between 65–34° N and 127–158° E [35,36]. Although wild cherry salmon populations have dramatically declined, the aquaculture of this species in freshwater has been sustained in Korea [37,38]. This species holds significant commercial value for both fisheries and aquaculture in Japan [39]. However, there have been relatively few studies conducted on the thermal biology of these fish [40,41,42,43]. Moreover, most existing studies on this species have relied on field surveys [42,43]. While a limited number of supplementary experiments have been conducted alongside these field studies to determine the effects of increasing temperature on the feeding response [41,42], both the acclimation period and the duration of the exposure were insufficient for the fish to physiologically respond to the changing conditions. Therefore, temperature experiments should be conducted in a controlled environment that includes a sufficient acclimation period. This will allow us to establish causative relationships between specific temperatures and physiological indices, including feeding and growth.

To the best of our knowledge, the present study represents the first attempt to isolate the specific effects of a range of temperatures typically encountered by juvenile cherry salmon in the land-based aquaculture farms of Korea. Therefore, the focal aim of the study is to conduct a comprehensive assessment, including growth performance, feeding utilization, RBC indices, stress indicators, and immune parameters, under controlled experimental conditions. Based on the assessment of the aforementioned parameters, the primary objectives encompassed identifying the temperature(s) conducive and less conducive to favorable conditions while assessing the adverse effects associated with the latter.

## 2. Materials and Methods

### 2.1. Fish Acquisition and Acclimation

For this study, commercially available cherry salmon parr (5 months old after hatching) from a farm in Hwacheon, Republic of Korea, were obtained and transported in oxygenated water to the indoor rearing facility at the Sejong Institute of Salmonid Research (SISR), Seoul, in June 2021. The fish were randomly netted and transferred in groups of 20 to each of 12 rectangular, recirculating, rearing tanks (glass, 300 L of water; each recirculating tank consisted of one rectangular rearing tank (120 × 45 × 45 cm) and one filtration tank (physical filtration, biological filtration, and sump sections; 120 × 45 × 45 cm)), which was equipped with a water chiller (DBA075, DAEIL, Busan, Korea) and/or heating bars with a thermos controller (KE-6422H, Sewon OKE, Seoul, Korea), and an additional air stone. The fish were acclimated to the tank conditions (temperature: 13.9 ± 0.07 °C; dissolved oxygen (DO) level: 9.63 ± 0.12 mg/L; pH: 7.66 ± 0.01, photoperiod: 14L/10D; conductivity: 737 ± 5.65; salinity: 0 ppt) for 2 weeks. During the acclimation period, the fish were fed a commercial trout diet (protein 51%, lipid 12%, ash 9%, and fiber 3%) twice a day (09:00 and 17:00) at a rate of 2.0% of their body weight (BW). About 20% of water from each tank was replaced with fresh dechlorinated and filtered tap water every day in the process of siphoning out the feces and uneaten feed.

### 2.2. Experimental Design and Sampling Procedure

After the 2-week acclimation period, four temperature treatments (10 °C, 14 °C, 18 °C, and 22 °C) were randomly assigned to the 12 tanks, resulting in three tanks for each treatment (n = 3). For the tanks that were assigned to 10 °C, the water in the tanks was gradually chilled (1 °C per day) to 10 °C using a water chiller (DBA075, DAEIL). In contrast, for the 18 °C and 22 °C temperature treatments, the water in the tanks was heated to 18 °C or 22 °C (1 °C per day) using heating bars with a thermos controller (KE-6422H, Sewon OKE, Seoul, Korea). Water temperatures for each tank were recorded twice a day using a multiparameter meter (Hi 9829, Hanna Instruments, Inc., Smithfield, RI, USA) and the multiparameter readings were verified with a mercury thermometer. After all of the tanks reached their target temperatures, the fish were acclimated to the temperatures for another 2 weeks before an 8-week growth trial. The growth trial was carried out between 2 July and 31 August 2021 (8 weeks) under a fixed photoperiod (14L:10D) in the indoor rearing facility of the SISR. During the 8-week growth trial, the juvenile salmon were fed a commercial trout diet (protein 51%, lipid 12%, ash 9%, and fiber 3%) twice a day (09:00 and 17:00) at a rate of 1.5% of their BW. Then, 30 min after feeding, the uneaten pellets were collected and counted to determine the feed intake and feed conversion ratio (FCR; Lee et al. [44]). Water quality was maintained as described in Table 1. DO, pH, and conductivity were measured daily using the multiparameter (Hi 9829, Hanna Instruments, Inc.,) and the multiparameter readings of DO and pH were verified with a bench top DO meter (LAQUA DO2000, Horiba Scientific, Tokyo, Japan) and pH meter (Lab PH meter F-71, Horiba Scientific, Tokyo, Japan), respectively. Total ammonia, nitrite, and nitrate were determined twice a week using a commercial kit (Test NH_3_/NH_4_^+^ Kit; Test NO_2_^−^ kit; Test NO_3_^−^, Tetra Spectrum Brands, LLC, Blacksburg, VA, USA).

All of the fish were fasted for 24 h before weighing and sampling and, at the beginning of the trial, all of the fish were individually weighed (average weight [g] ± SE: 29.1 ± 0.19) and their fork length (average fork length [cm] ± SE: 13.4 ± 0.05) was measured. After 4 and 8 weeks, 8 fish and 12 fish, respectively, from each tank were randomly captured and then euthanized using MS-222 (300 mg/L; pH 7.5; Sigma-Aldrich, St. Louis, MO, USA). They were individually weighed and their fork length was measured to determine the growth parameters. From three individuals among the euthanized fish at each sampling time, blood was collected from the caudal vein using a syringe and heparinized tubes (lithium heparin tube, BD, Franklin Lakes, NJ, USA) [45]. Then, a part of the whole blood was used to determine the Hct, RBC count, and Hb concentration of the blood as previously described [45], and the mean corpuscular volume (MCV), mean corpuscular hemoglobin (MCH), and mean corpuscular hemoglobin concentration (MCHC) were calculated as described in Section 2.3 [44]. Next, the plasma was separated from the rest of the blood after centrifugation at 13,000× *g* and 4 °C (Centrifuge Smart R17 Plus, Hanil Scientific Inc., Gimpo, Korea) for 15 min, and it was stored at −80 °C for later use.

After the blood collection, the gill filaments from the same fish were removed using scissors, put into an ice-cold SEI buffer, immediately frozen using dry ice, and stored at −80 °C for the determination of the Na^+^/K^+^-ATPase activity. Subsequently, the liver from the same fish was surgically removed and weighed to determine the hepatosomatic index (9 fish per treatment; 3 fish per tank). At the end of the growth trial, the same sampling procedure was conducted as the one used in week 4, except that 12 fish were sampled rather than 8. The study was conducted according to the protocol approved by the Animal Care and Use Committee of Sejong University (Protocol and approval date: SJ20210711 and 21 January 2021).

### 2.3. Calculations

The following formulae were used to calculate the growth parameters, FCR, and daily individual feed intake [44]:

SGR = 100 × [ln (final BW) − ln (initial BW)]/days

Percentage of weight gain (%WG) = 100 × (final fish weight − initial fish weight)/initial fish weight

CF = 100 × BW (g)/body length^3^ (cm^3^)

HSI = 100 × liver weight (g)/fish BW (g)

FCR = feed intake (g)/BW gain (g)

Daily individual FI (g/d) = g feed consumed/number of days/number of fish

MCV = Hct (%) × 100/RBC count (10^6^ × mm^−3^)

MCH = Hb (g/dL) × 10/RBC count (10^6^ × mm^−3^)

MCHC = Hb (g/dL) × 100/Hct (%)

where SGR is the specific growth rate, %WG is the percentage weight increase per day, CF is the condition factor, HSI is the Hepatosomatic index, Hct is the hematocrit, and Hb is hemoglobin.

### 2.4. Determination of the Plasma Cortisol and Glucose Levels

The plasma cortisol concentration was individually determined (9 fish per treatment) using an enzyme-linked immunosorbent assay (ELISA) kit (Fish Cortisol ELISA kit, CUSABIO, Houston, TX, USA) as previously described [44]. Briefly, a competitive (inhibition) reaction between the pre-coated cortisol on the bottom of the well in a 96-well plate and the cortisol in plasma samples and the standard was used to quantify cortisol in the plasma samples using a calibration (standard) curve. A substrate was added to the wells and the color developed according to the cortisol amount in the samples, which was measured at 450 nm in a microplate reader (Epoch 2 Microplate Reader, BioTek Instrument, Winooski, VT, USA). Each sample was determined in duplicate. A standard curve was constructed using a cortisol standard (10 ng/mL) that was provided in the kit to back-calculate the cortisol concentration in the plasma samples from the color intensity. No significant cross-reactivity or interference between fish cortisol and analogs was observed according to manufacturer’s protocol, and the detection limit was 0.0023 ng/mL. Additionally, the plasma glucose concentration was determined using the glucose oxidase–peroxidase method using a clinical glucose assay kit (AM 201-K, Asan Pharm, Seoul, Korea).

### 2.5. Immunoglobulin M and Lysozyme Activity

The plasma immunoglobulin M (IgM) level was individually determined (9 fish per treatment) using a commercial ELISA assay kit (Fish IgM ELISA kit, CUSABIO, Houston, TX, USA) based on a competitive inhibition reaction between horseradish peroxidase (HRP)-labeled IgM and unlabeled IgM in the samples with a pre-coated antibody [44]. Each assay was conducted in duplicate, and the color development was measured at 450 nm and 37 °C. The detection range was 1.25–50 mg/mL.

The plasma lysozyme content was also individually determined (9 fish per treatment) using an ELISA assay kit (Fish Lysozyme ELISA kit, CUSABIO, Houston, TX, USA). Briefly, the assay protocol was based on the competitive inhibition enzyme immunoassay technique between the lysozyme in the sample and the provided HRP-conjugated lysozyme in the wells with a pre-coated antibody specific to lysozymes. Each sample was analyzed in duplicate, and the color intensity was determined at 450 nm with the correction wavelength at 540 nm and 37 °C. Moreover, the detection range was 3.12–200 ng/mL.

### 2.6. Sodium/Potassium-Activated Adenosine Triphosphatase Activity

The gill Na^+^/K^+^-ATPase activity was determined based on the method by McCormick [46] and a detailed procedure is described in the article [44]. Each assay was conducted in duplicate on individual gill samples from each fish (9 fish per treatment). Briefly, the protocol involved measuring the ouabain-sensitive hydrolysis of adenosine triphosphatase (ATP) and the subsequent decrease in nicotinamide adenine dinucleotide oxidation by pyruvate kinase and lactate dehydrogenase. The measurement was taken at a wavelength of 340 nm and a temperature of 25 °C for 10 min using a microplate reader (Epoch 2 microplate reader, BioTek Instrument, Santa Clara, CA, USA). The Na^+^/K^+^- ATPase activity was calculated by comparing the ATP hydrolysis levels in the presence and absence of ouabain, and the result was expressed as micromoles of adenosine diphosphate per milligram of protein per hour. Furthermore, the assays were conducted in duplicate to ensure reliability.

### 2.7. Statistical Analysis

The tank was used as a sampling unit (n = 3). Three fish per tank were sampled (nine fish per treatment) at each sampling time point for all biochemical assays, including cortisol, glucose, gill Na^+^/K^+^- ATPase activity, lysozyme, and IgM determination. The data were statistically analyzed using SPSS Statistics 21.0 (IBM, Chicago, IL, USA). A two-way repeated measures ANOVA was performed to assess the effects of water temperature and exposure time on the growth and feed utilization parameters, RBC indices (except Na^+^ level), stress, and immune parameters. In cases where the assumptions of the two-way repeated measures ANOVA were not met, appropriate statistical corrections were applied and detailed in the results. Additionally, a one-way ANOVA was performed on the plasma Na^+^ concentration and gill Na^+^/K^+^-ATPase activity. To further explore differences among treatments and between time points, pair-wise comparisons were conducted using Duncan’s Multiple Range Test. A significance level of *p* < 0.05 was used to define statistical significance, and the data were presented as the mean ± standard error of the mean.

## 3. Results

### 3.1. Growth Performance

After 4 weeks and 8 weeks of the growth trial, the different temperatures had significant effects on the growth parameters (FL, BW, SGR, %WG, and CF) of the juvenile cherry salmon (*p* < 0.05, Table 2). At week 4 and week 8, the FLs of the salmon were significantly higher at 10 °C and 14 °C when compared with those at 18 °C and 22 °C (*p* < 0.05, Table 2). At both time points, the FL at 22 °C was significantly lower than that at 18 °C (*p* < 0.05; Table 2). At week 4, the total BWs at 10 °C and 14 °C were significantly higher when compared with those at 18 °C and 22 °C (*p* < 0.05, Table 2). At week 8, the BWs at 10 °C and 14 °C were significantly higher than those at 18 °C and 22 °C (*p* < 0.05, Table 2), and the BW at 22 °C was significantly lower than that at 18 °C (*p* < 0.05, Table 2).

Both the SGR and %WG at 10 °C were not significantly different from those at 14 °C at both week 4 and week 8 (*p* > 0.05, Table 2). At week 4, both the SGR and %WG at 18 °C were significantly lower than those at 10 °C and 14 °C but higher than that at 22 °C (*p* < 0.05, Table 2). After 8 weeks, both the SGR and %WG at 18 °C and 22 °C were significantly lower than those at 10 °C and 14 °C (*p* < 0.05, Table 2). However, at week 8, the SGR and %WG at 18 °C were not significantly different from those at 22 °C (*p* > 0.05, Table 2).

The CF at week 4 and week 8 exhibited significant gradual increases with increasing temperature (*p* < 0.05, Table 2). There was no significant difference in HSI among treatments and between time points (*p* > 0.05, Table 2). The different temperature treatments did not result in any mortality during the trial.

### 3.2. Feed Conversion Ratio and Daily Individual Feed Intake

The FCR at 10 °C was not significantly different from those at 14 °C at both week 4 and week 8 (*p* > 0.05, Table 2). At week 4, the FCR at 18 °C was significantly higher than that at 10 or 14 °C but significantly lower than that at 22 °C (*p* < 0.05, Table 2). At week 8, the FCR at 18 °C or 22 °C was significantly higher than that at 10 °C or 14 °C (*p* < 0.05, Table 2), while the FCR at 18 °C was lower than that at 22 °C without statistical significance (*p* > 0.05, Table 2).

There were significant effects of temperature, time, and their interaction on the FI (*p* < 0.05, Table 2). At week 4, there was no significant difference among temperature treatment groups (*p* > 0.05, Table 2). However, at week 8, the FI at 18 °C or 22 °C was significantly lower than that at 10 °C or 14 °C (*p* < 0.05, Table 2). Additionally, the FI at 22 °C was lower than that at 18 °C without statistical significance (*p* > 0.05, Table 2).

### 3.3. Red Blood Cell Indices

At week 4, the Hct of the cherry salmon at 18 °C and 22 °C were significantly lower than that at 10 °C (*p* < 0.05, Table 3). However, at week 8, the Hct at 22 °C was lower than that at 10 °C, 14 °C, or 18 °C without statistical significance (*p* > 0.05, Table 3). At week 4, the RBC count at 22 °C was lower than that at 10 °C, 14 °C, or 18 °C without statistical significance (*p* > 0.05, Table 3). However, at week 8, the RBC count at 22 °C was significantly lower than that at 10 °C, 14 °C, or 18 °C (*p* < 0.05, Table 3), and the RBC count gradually decreased with increasing temperatures (Table 3). The plasma hemoglobin concentration (g/dL) at 22 °C was lower than those at the other temperatures at week 8, but the difference was not statistically significant (*p* > 0.05, Table 3). Additionally, while only the MCH was significantly higher at 22 °C than those at the other temperatures at week 4 (*p* < 0.05, Table 3), both the MCV and MCH were significantly higher at 22 °C than those at the other temperatures at week 8 (*p* < 0.05, Table 3). However, there was no significant effect of temperature on plasma osmolality, MCHC (*p* > 0.05; two-way repeated measure ANOVA), and Na^+^ concentration (one-way ANOVA) (Table 3).

### 3.4. Plasma Cortisol and Glucose

At week 4, the plasma cortisol concentration at 10 °C was significantly higher than at 22 °C (*p* < 0.05, Table 4). At week 8, the cortisol concentration at 10 °C was significantly higher than at 18 °C or 22 °C (*p* < 0.05, Table 4). Regarding plasma glucose levels, the homogeneity of variance of the differences (the assumption of sphericity) among groups was violated, so the analysis was corrected using Greenhouse–Geisser correction methods. However, temperature did not significantly affect plasma glucose concentrations (*p* > 0.05, Table 4). Nine fish were individually analyzed per treatment for all biochemical assays, as well as cortisol and glucose levels, at each time point.

### 3.5. Gill Na^+^/K^+^- Adenosine Triphosphatase (ATPase) Activity

The gill Na^+^/K^+^-ATPase activity significantly lowered with increasing temperature at week 8 (*p* < 0.05, Table 4). Specifically, the gill Na+/K+-ATPase activity at 10 °C was significantly higher than those at the other temperatures (*p* < 0.05). In addition, the gill Na^+^/K^+^-ATPase activity at 14 °C or 18 °C was significantly higher than that at 22 °C (*p* < 0.05). However, there was no significant difference in the gill Na^+^/K^+^-ATPase activity between 14 °C and 18 °C (*p* > 0.05). The analysis of gill Na^+^/K^+^-ATPase activity at week 4 was not conducted due to accidental sample loss.

### 3.6. Plasma Immunoglobulin M and Lysozyme Activity

Temperature did not significantly impact plasma IgM concentrations at both time points (*p* > 0.05; two-way repeated measures ANOVA, Table 4). At both weeks 4 and 8, the plasma lysozyme concentrations at 22 °C were significantly higher when compared with those at 10 °C, 14 °C, or 18 °C (*p* < 0.05, Table 4).

## 4. Discussion

This study represents the first controlled experiment examining the effect of temperatures that are commonly found in commercial aquaculture systems in South Korea on growth, feed utilization, stress, and hemato-immune responses in juvenile cherry salmon. This study demonstrated that temperatures of 10 °C and 14 °C were the most favorable within the designated temperature range for growth and feed performances in juvenile cherry salmon under the experimental settings. Conversely, temperatures of 22 °C or higher had detrimental effects on various physiological functions of the cherry salmon, such as RBC maintenance, feeding response, appetite, immune parameters, smoltification, and growth.

Our results demonstrated that juvenile cherry salmon exhibited the highest growth at temperatures of 10 °C and 14 °C, and they were able to survive and grow at continuously increasing temperatures up to 22 °C during their freshwater growth life stage. However, we observed that the elevated temperatures, specifically 18 °C and 22 °C, had an impact on the growth (rate), feed intake, and feed efficiency of the fish. Similar findings regarding the effects of temperature on growth have been reported in previous studies on other salmonid species [2,3,5,7]. For example, sockeye salmon exhibited optimal growth at 15 °C, while they exhibited no growth at around 23 °C [2]. In juvenile Sacramento River Chinook salmon (*Oncorhynchus tshawytscha* Walbaum), better growth, appetite, and smolt physiology were observed in the temperature range of 13–16 °C when compared with those at the higher temperature range of 21–24 °C [3]. Juvenile Atlantic salmon exhibited the highest growth rate at 14–15.6 °C when compared with those at other temperatures [5,7]. In cherry salmon, previous studies have explored the effects of temperature on growth and feeding [41,42,43]. For example, Sato et al. [42] and Morita and Nagasawa [43] found a positive correlation between growth and stream water temperature up to 24 °C based on their field survey. The overall finding of the previous studies of an upper-temperature limit (24 °C) for growth does not contradict this study’s results.

In this study, the feed intake reached its peak at 14 °C and subsequently decreased at 18 °C and 22 °C. Similarly, Brett et al. [2] reported decreased food intake in sockeye salmon fingerlings above approximately 17–18 °C, with a loss of appetite observed at around 22–23 °C. Similar observations were made in Atlantic salmon and cherry salmon, where feed intake or appetite decreased above ~18 °C [6,7,40,47,48]. Consequently, temperatures of approximately 18 °C or higher could potentially hinder the feeding behavior of cherry salmon. In contrast, there appears to be a discrepancy in the tipping temperature for appetite decrease between the results of the current study and Sato et al. [42] and Takami and Sato [41], which observed decreases in appetite above 24 °C in short-term feeding experiments. One possible explanation is that the durations of the studies (6 days and 12 days, respectively), including the acclimation periods, were significantly shorter than that of this study. These shorter durations may not have allowed for enough time for the salmon to complete their biochemical and physiological transformation during the acclimation process [49].

The decreased feed efficiencies observed at the temperatures of 18 °C and 22 °C in this study have been previously reported at elevated temperatures in other studies [30,47,50]. These studies have shown that the effects of temperature increments on feed efficiency are dependent on the ration level. Specifically, at a lower ration level, elevated temperatures have been found to decrease feed efficiency in various fish species, including salmonids. This decline in efficiency can be attributed to the increased standard metabolic rate (SMR) that accompanies temperature increases within physiologically permissive ranges in fish [6,30,50]. Consequently, the increased SMR at elevated temperatures reduces the energy available for growth, resulting in reduced feed efficiency and growth [30,47,51]. Therefore, the observed decrease in feed efficiency at the elevated temperatures of 18 °C and 22 °C in this study can be attributed, at least partially, to the increased SMR that is associated with those temperatures. Furthermore, it can be speculated that the decreased feed intake at high temperatures may be related to the limited aerobic scope and a preparatory appetite reduction to conserve energy for vital activities in a possible state of negative energy balance at the given ration size of this study [30,47]. Taken together, a presumably increased SMR and the observed decrease in FI at high temperatures are the primary factors responsible for the reduced feed efficiency and growth observed in this study.

The decreased Hct, RBC count, and Hb and increased MCV and MCH at the elevated temperature (22 °C) in this study are consistent with findings from previous studies [12,13]. Langston et al. [13] attributed the lowest packed cell volume at 18 °C to thermal stress, when compared with the highest at 12 °C and 15 °C, in Atlantic halibut (*Hippoglossus* L.) that were acclimated at four temperatures (8 °C, 12 °C, 15 °C, and 18 °C) for three months. Also, Lochmiller et al. [12] attributed substantially decreased Hct, RBC, and Hb levels in striped bass (*Morone saxatilis* Walbaum) during the summer, in comparison with those in the other seasons, to secondary nutritional problems caused by heat stress and low oxygen levels. They supported this hypothesis of erythropoietic depression due to nutritional problems by citing the elevated MCV and MCH values observed during the summer. On the other hand, Martinez et al. [11] attributed the increased Hct, RBC, and Hb levels at 20 °C compared to those at 15 °C to a compensatory increase in RBCs to meet the increased metabolic demand under normoxic conditions. Similarly but differently, the decreased FI in the current study, coupled with an expected increase in SMR and decrease in aerobic scope, could contribute to the depression of hematopoietic processes [12,30]. This depression might have led to decreased Hct, RBC count, and Hb levels, as well as increased MCV and MCH at high temperatures. In addition, evidence of hemodilution or hemoconcentration was not observed based on the plasma osmolality and Na+ concentration.

In the current study, the plasma cortisol levels decreased with increasing temperatures from 10 °C to 22 °C, contrary to our initial expectations. A possible explanation for the higher plasma cortisol levels at 10 °C in week 8 than that in week 4 could be related to the hormonal change that is associated with smolt development [52,53]. The lower cortisol levels at 14 °C, 18 °C, and 22 °C, when contrasted with that at 10 °C in week 8, may indicate the suppression of smolt development or a more rapid reversion to a pre-smolt condition at the temperatures of 14 °C, 18 °C, and 22 °C in cherry salmon, as previously reported at elevated temperatures in other anadromous salmonids [1,28,29,53]. Cortisol is a major seawater-adapting hormone, as experimental cortisol infusion has been shown to enhance seawater adaptation and tolerance [26,52,54]. Previous studies have reported that plasma cortisol levels increase before and during parr–smolt transformation in salmonids and correlates with the induction of the hypo-osmoregulatory capacity in preparation for seawater survival [52,54]. However, temperatures above a certain threshold have been found to hinder the normal development of hypo-osmoregulatory ability in salmonids, which were experimentally proved in Atlantic salmon smolts above 20 °C [29], steelhead trout at 13 °C or higher [28], and in Chinook salmon smolts above 17 °C [1]. On the other hand, elevated temperatures are also known to accelerate desmoltification in anadromous salmonids, including coho salmon (*Oncorhynchus kisutch*) and Chinook salmon, leading to rapid reversion to a pre-smolt condition [55].

The present study observed an increase in the body silvering of juveniles at 10 °C and a decrease with rising temperatures at week 8, significant decreases in the CF with decreasing temperatures when compared with that at higher temperatures (Table 2), and a significant increase in the Na^+^/K^+^-ATPase activity at 10 °C when compared with those at 14 °C, 18 °C, or 22 °C at week 8 (Table 4). Similarly, McCormick [53] reported that these parameters, along with the increased plasma cortisol levels, are distinct characteristics that are associated with parr–smolt transformation.

Juvenile anadromous salmon in freshwater undergo morphological, behavioral, and physiological changes in preparation for survival in seawater [53]. The crucial aspect of this transformation is the acquisition of ion regulation capacity in hyperosmotic environments, such as seawater, with Na^+^/K^+^-ATPase in the chloride cells of the gills playing a major role. However, as mentioned earlier, elevated temperatures are known to accelerate desmoltification, thus hastening reversion to a pre-smolt condition that includes Na^+^/K^+^-ATPase activity in anadromous salmonids [55,56]. Zaugg and Mclain [56] showed that elevated temperatures hastened the reversion of gill Na^+^/K^+^-ATPase activity to a pre-smolt level in coho salmon. Furthermore, previous studies have shown that high temperatures can disrupt normal transformation by inhibiting the stimulation of gill Na^+^/K^+^-ATPase activity [28,29]. Zaugg and Wagner [28] showed that Na^+^/K^+^-ATPase activity in steelhead trout that were exposed to temperatures of 13 °C or higher was significantly reduced. Furthermore, Marine and Cech [1] also reported significantly depressed gill Na^+^/K^+^-ATPase activity in the group that was exposed to temperatures of 21–24 °C in comparison with that of the group that was exposed to temperatures of 13–16 °C in Chinook salmon smolts.

Although the cherry salmon in this study were under-yearlings, given that the fish showed good growth (above the threshold size limits) [57] (Table 2) under the favorable environmental settings (as shown in Table 1) with an adequate feed supply, developmental cues might have prompted smolt development for seawater readiness at favorable temperatures, such as the temperatures of 10 °C or 14 °C used in this study, as previously described [53]. Therefore, the significantly higher Na^+^/K^+^-ATPase activity at 10 °C may imply the preferred or ecological temperature(s) at which this species is likely to inhabit or favor in its natural habitat at this developmental stage.

The increased lysozyme concentration at the highest temperature in this study was attributed to enhanced lysozyme activity or factors, such as the presence of pathogens, stress, or environmental conditions like temperature in previous studies [13,58,59]. Given the absence of apparent signs or indications of infection among the studied fish, our discussion focused on alternative contributing elements. Previous studies reported that environmental stressors can increase lysozyme activity in diverse fish species, including salmonids [59,60,61,62]. Langston et al. [13] further reported that lysozyme activity increased in a temperature-dependent manner between 8 °C and 18 °C in some strains of Atlantic halibut (*Hippoglossus hippoglossus* L.). They described the increased lysozyme activity at the elevated temperature due to stress based on blood parameters, without the inclusion of stress biomarkers such as plasma cortisol and glucose [13]. Moreover, some studies reported that Chinook salmon and Atlantic salmon, subject to chronic exposure to higher temperatures (21 °C and 20 °C), did not show significant increases in cortisol or glucose levels [24,63]. Collectively, while we did not detect significant cortisol and glucose increases at the sampling time points in the current study, it remains plausible that the observed increase in plasma lysozyme levels might be correlated with responses to thermal stress at the high temperature (22 °C).

## 5. Conclusions

Based on our experimental settings, the most suitable temperatures for the growth and feed performances and physiological parameters fall within the range of 10 °C to 14 °C for juvenile cherry salmon (approximately 29 g in mass). Interestingly, all the parameters related to smoltification, such as dermal silvering, the CF, the cortisol level, and Na^+^/K^+^-ATPase activity, suggest that a temperature of around 10 °C is most favorable for smoltification development within the experimental temperature range. However, a temperature of 14 °C, rather than 10 °C, might be a more suitable option for both the fish’s growth performance and physiological conditions. This is because all determined parameters, including growth, feed utilization, and health, at 14 °C were comparable with those at 10 °C with significantly reduced smoltification parameters. This reduction could be more advantageous for the freshwater production of this species.

Even though there were no observed positive changes in plasma stress responses (cortisol and glucose) at this temperature, all of the endpoints suggest that 22 °C is above the physiologically preferred thermal range. Furthermore, prolonged exposure to that temperature may potentially impact the health, normal physiological development, and overall well-being of juvenile cherry salmon. This is supported by the observed decrease in RBC indices, smoltification-related parameters, disturbed immune parameters, and depressed feeding response and appetite noted in the current study.

Consequently, further validation is needed in future studies to determine whether the significant changes in growth, feed efficiency, and RBC parameters at high temperatures are primarily attributed to chronic thermal stress, increased (standard) metabolic rate leading to elevated biochemical reaction rates, or other alterations in feeding and digestive processes. Future research that includes the investigation of additional immune parameters and stress biomarkers will contribute to a better understanding of the underlying mechanism of the thermal biology in this species. The study’s insights into the temperature range (10 °C to 14 °C) crucial for cherry salmon physiology and smoltification have direct implications for their conservation in natural habitats. Understanding these temperature impacts aids in formulating targeted conservation strategies aligned with the species’ preferences. Moreover, recognizing adverse effects at temperatures of 22 °C or higher highlights the urgency to mitigate climate-induced threats. A comprehensive approach, including immune responses and metabolic dynamics, is essential for refining conservation measures to safeguard cherry salmon populations in their native ecosystems.

## Figures and Tables

**Table 1:** Water quality parameters and photoperiod.

Parameter	Treatment			
	10 °C	14 °C	18 °C	22 °C
Water temperature (°C)	10.30 ± 0.05	14.15 ± 0.04	18.11 ± 0.05	21.97 ± 0.04
Photoperiod (L:D) Dissolved oxygen (mg/L)	14:10 10.66 ± 0.11	14:10 9.34 ± 0.08	14:10 8.00 ± 0.09	14:10 7.98 ± 0.08
pH	7.72 ± 0.02	7.66 ± 0.01	7.61 ± 0.02	7.69 ± 0.02
Conductivity (mS/cm)	710.17 ± 7.35	733.50 ± 8.29	758.26 ± 9.72	755.3 ± 8.67
Ammonia (mg/L) ^†^	Not detected	Not detected	Not detected	Not detected
Nitrite (mg/L) ^‡^	Not detected	Not detected	Not detected	Not detected
Nitrate (mg/L)	13.25 ± 0.75	16.56 ± 1.48	16.32 ± 1.41	15.36 ± 1.31

Data are presented as mean ± standard error. **^†^** Detection limit was 0.25 mg/L. **^‡^** Detection limit was 0.3 mg/L.

**Table 2:** Growth and feed performances of cherry salmon (*Oncorhynchus masou*) exposed to four temperatures for 4 and 8 weeks.

		FL (cm)	BW (g)	SFG (%BW/d)	%WG	CF	HSI	FCR	FI (g/d)
Initial		13.44 ± 0.05	29.11 ± 0.19			1.19 ± 0.01	1.78 ± 0.09		
Week 4	10 °C	14.95 ± 0.18 d	37.66 ± 1.43 b	0.93 ± 0.04 c	29.73 ± 1.53 cd	1.12 ± 0.01 a	1.84 ± 0.17	1.10 ± 0.05 a	0.34 ± 0.004 a
	14 °C	14.80 ± 0.15 cd	38.87 ± 1.22 b	0.96 ± 0.02 c	30.76 ± 0.76 d	1.19 ± 0.01 bc	1.56 ± 0.07	1.07 ± 0.03 a	0.35 ± 0.002 a
	18 °C	14.18 ± 0.19 b	35.18 ± 1.44 a	0.76 ± 0.07 b	23.74 ± 2.57 bc	1.22 ± 0.02 cd	1.66 ± 0.05	1.42 ± 0.15 b	0.34 ± 0.005 a
	22 °C	13.81 ± 0.18 a	33.74 ± 1.96 a	0.52 ± 0.02 a	15.69 ± 0.63 a	1.25 ± 0.03 cd	1.82 ± 0.02	2.11 ± 0.08 d	0.34 ± 0.004 a
Week 8	10 °C	16.55 ± 0.14 e	52.41 ± 1.58 d	1.03 ± 0.06 c	50.80 ± 3.72 e	1.14 ± 0.01 ab	1.56 ± 0.05	1.00 ± 0.08 a	0.48 ± 0.02 d
	14 °C	16.08 ± 0.19 e	51.24 ± 2.09 d	0.99 ± 0.04 c	48.91 ± 1.85 e	1.21 ± 0.01 c	1.48 ± 0.03	1.05 ± 0.04 a	0.50 ± 0.001 d
	18 °C	15.02 ± 0.21 d	42.77 ± 1.96 c	0.60 ± 0.04 a	26.32 ± 1.45 bcd	1.23 ± 0.01 cd	1.65 ± 0.25	1.79 ± 0.11 c	0.44 ± 0.009 c
	22 °C	14.55 ± 0.16 bc	40.53 ± 1.82 b	0.51 ± 0.02 a	20.3 ± 0.91 ab	1.28 ± 0.01 d	1.90 ± 0.20	2.06 ± 0.15 cd	0.41 ± 0.030 b

Fork length—FL; body weight—BW; specific growth rate—SGR; % weight gain—%WG; condition factor—CF; hepatosomatic index—HSI; feed conversion ratio—FCR; individual feed intake—FI. Dissimilar letters indicate significantly different groups among temperature groups and between time points at a probability of 0.05. Values are means ± standard errors of the mean (8–12 fish per replicate for growth parameters; 12–20 fish per replicate for feed parameters).

**Table 3:** Hematological indices of cherry salmon (*Oncorhynchus masou*) exposed to four temperatures for 4 and 8 weeks.

		Plasma Osmolality (mOsmol/kg)	Plasma Na^+^ Level (mM)	Hematocrit (%)	RBC Count (× 10^6^ mm^−3^)	Hemoglobin (g/dL)	MCV (fL)	MCH (pg)	MCHC (g/dL)
Initial				37.96 ± 1.29	2.26 ± 0.13	9.80 ± 0.22	172.18 ± 10.05	44.69 ± 2.89	25.91 ± 0.34
Week 4	10 °C	307.0 ± 2.59	NA	40.87 ± 1.61 b	2.43 ± 0.12 abc	10.48 ± 0.31	169.57 ± 5.84 bc	43.65 ± 1.73 a	25.75 ± 0.57
	14 °C	307.8 ± 3.26	NA	37.38 ± 0.88 ab	2.41 ± 0.09 abc	10.27 ± 0.16	156.24 ± 3.89 ab	43.27 ± 2.11 a	27.63 ± 0.93
	18 °C	302.4 ± 2.06	NA	37.10 ± 0.98 ab	2.43 ± 0.15 abc	10.38 ± 0.25	156.05 ± 7.15 ab	43.57 ± 1.66 a	28.02 ± 0.38
	22 °C	308.0 ± 3.65	NA	35.59 ± 1.37 a	2.13 ± 0.17 ab	10.38 ± 0.31	174.4 ± 12.73 bc	51.27 ± 4.26 b	29.27 ± 0.54
Week 8	10 °C	308.3 ± 2.00	155.9 ± 0.80	37.48 ± 1.52 ab	2.75 ± 0.11 c	10.31 ± 0.27	136.75 ± 3.35 a	37.77 ± 0.98 a	27.67 ± 0.58
	14 °C	304.0 ± 4.30	152.8 ± 0.87	36.06 ± 1.02 a	2.61 ± 0.16 bc	10.18 ± 0.24	140.64 ± 5.31 a	39.72 ± 1.46 a	28.27 ± 0.28
	18 °C	306.8 ± 2.29	153.3 ± 1.86	37.98 ± 1.40 ab	2.51 ± 0.14 bc	10.43 ± 0.27	153.09 ± 6.10 ab	42.2 ± 1.77 a	27.67 ± 1.00
	22 °C	310.3 ± 3.75	154.3 ± 1.11	35.13 ± 1.44 a	1.98 ± 0.14 a	9.86 ± 0.27	182.38 ± 10.23 c	51.67 ± 3.53 b	28.22 ± 0.62

Red blood cell—RBC; mean corpuscular volume—MCV, mean corpuscular hemoglobin—MCH; mean corpuscular hemoglobin concentration—MCHC. Dissimilar letters indicate significantly different groups among temperature groups and between time points at a probability of 0.05. Values are means ± standard errors of the mean (3 fish per replicate; 9 fish per treatment).

**Table 4:** Plasma stress, gill Na^+^/K^+^-ATPase activity, and humoral immune response of cherry salmon (*Oncorhynchus masou*) exposed to four temperatures for 4 and 8 weeks.

		Cortisol (ng/mL)	Glucose (mg/dL)	Na^+^/K^+^-ATPase Activity (μmoles ADP/mg Protein/hour)	Immunoglobulin M (mg/mL)	Lysozyme (ng/mL)
Week 4	10 °C	2.26 ± 0.71 b	76.99 ± 4.46	NA	6.32 ± 1.56	97.53 ± 16.33 a
	14 °C	1.00 ± 0.19 ab	64.41 ± 2.20	NA	7.44 ± 1.68	97.47 ± 15.93 a
	18 °C	0.49 ± 0.18 ab	70.74 ± 1.43	NA	5.72 ± 1.39	104.58 ± 15.48 a
	22 °C	0.33 ± 0.11 a	66.69 ± 1.74	NA	5.59 ± 0.84	175.93 ± 10.34 b
Week 8	10 °C	4.45 ± 1.25 c	72.91 ± 2.21	2.11 ± 0.29 a	7.75 ± 1.60	100.00 ± 16.42 a
	14 °C	2.61 ± 0.95 bc	68.18 ± 2.70	1.39 ± 0.16 b	4.62 ± 1.19	105.31 ± 11.03 a
	18 °C	1.04 ± 0.46 ab	73.52 ± 2.44	1.35 ± 0.11 b	9.74 ± 1.63	105.20 ± 12.08 a
	22 °C	0.67 ± 0.15 ab	75.59 ± 2.24	0.73 ± 0.12 c	11.51 ± 2.18	182.82 ± 9.26 b

Dissimilar letters indicate significantly different groups among temperature groups and between time points at a probability of 0.05. Values are means ± standard errors of the mean (3 fish per replicate; 9 fish per treatment). Not available due to the limited blood volume—NA.

## Data Availability

The data presented in this study are available on request from the corresponding authors.
